# Retroperitoneal and Pelvic Myeloid Sarcoma in a Patient With JAK2-Positive Polycythemia Vera: A Case Report

**DOI:** 10.7759/cureus.110684

**Published:** 2026-06-11

**Authors:** Mozhan M Hagigatian, Joel Thompson, Mohammad Hussain, Joseph Hatem, Fadi J Hatem

**Affiliations:** 1 Radiology, Lake Erie College of Osteopathic Medicine, Erie, USA; 2 Radiology, Rochester Regional Health, Rochester, USA; 3 Pathology, Rochester Regional Health, Rochester, USA

**Keywords:** chloroma, extramedullary leukemia, granulocytic sarcoma, myeloid sarcoma, polycythemia vera

## Abstract

Myeloid sarcoma (MS) is a rare extramedullary tumor composed of immature myeloid cells that may occur in association with acute myeloid leukemia or other myeloid neoplasms. We report a 75-year-old woman with JAK2-positive polycythemia vera who presented with abdominal pain, distension, and anorexia. Imaging revealed a large retroperitoneal and pelvic mass causing hydronephrosis. Biopsy demonstrated immature mononuclear cells positive for CD33, CD43, CD117, CD68, and CD163, consistent with MS with monocytic differentiation. Other malignancies were excluded, and flow cytometry was non-diagnostic. This case highlights the diagnostic challenge of MS and underscores the importance of integrating imaging, histopathology, and immunophenotyping for accurate diagnosis. Early recognition is critical, as untreated MS can progress to acute leukemia. Clinicians should consider MS in patients with myeloproliferative neoplasms presenting with atypical masses to guide timely therapy and improve outcomes.

## Introduction

Myeloid sarcoma (MS), formerly known as granulocytic sarcoma or chloroma, is a rare extramedullary tumor composed of immature myeloid cells and is most associated with acute myeloid leukemia (AML), although it may also occur in patients with myeloproliferative neoplasms (MPNs) or myelodysplastic syndromes [1,2]. MS occurs in approximately 2.5-9% of patients with AML, whereas isolated MS without bone marrow involvement is exceedingly uncommon, with an estimated incidence of approximately two cases per million adults [1,3]. The disease may present concurrently with AML, precede the diagnosis of leukemia, or arise as an isolated lesion in the absence of detectable bone marrow involvement [1,4].

MS can involve a wide range of anatomic sites, including lymph nodes, skin, soft tissue, bone, testes, peritoneum, and the gastrointestinal tract [1,5]. Retroperitoneal and pelvic involvement is uncommon and may mimic other soft tissue neoplasms or metastatic malignancies [3,6]. As a result, clinical symptoms vary considerably depending on the site of involvement. Imaging findings are often non-specific, making histopathologic examination and immunophenotypic characterization essential for establishing the diagnosis.

Due to its rarity and variable presentation, diagnosis is often challenging and may be delayed. Patients frequently present with symptoms related to mass effect rather than hematologic abnormalities, and MS may be mistaken for lymphoma, sarcoma, or other solid tumors. Early recognition is important because isolated MS frequently progresses to systemic leukemia if left untreated [1,4].

While MS is well documented in connection with de novo AML, its occurrence in polycythemia vera (PV) is extremely rare, mostly appearing in isolated case reports and small studies. In patients with MPNs, the development of MS may represent an extramedullary manifestation of disease acceleration or blast transformation, even in the absence of overt bone marrow blast-phase disease [7]. Recent WHO and International Consensus Classification (ICC) frameworks recognize the close biological relationship between advanced-phase MPNs and secondary AML arising from antecedent MPNs, emphasizing the importance of identifying extramedullary blast proliferation in this clinical setting [8,9].

We present a case of retroperitoneal and pelvic MS in a patient with JAK2-positive PV, highlighting the diagnostic challenges associated with this unusual presentation.

## Case presentation

A 75-year-old woman with a known history of PV, positive for a JAK2 mutation, on aspirin and hydroxyurea, presented with a two-week history of intermittent, generalized abdominal pain associated with cramping, bloating, nausea, and dry heaving. She was unable to identify any precipitating or relieving factors. The patient also reported progressive anorexia, which worsened to the point of being unable to tolerate oral intake.

Review of systems was negative for fever, chills, shortness of breath, hemoptysis, chest pain, diarrhea, hematochezia, syncope, unintentional weight loss, fatigue, night sweats, recent travel, or sick contacts. She had a 40-pack-year smoking history and currently smoked six to eight cigarettes per day.

On physical examination, the abdomen was distended with generalized tenderness to deep palpation, and the left costovertebral angle tenderness was noted. Initial laboratory evaluation revealed a white blood cell count of 10.4 × 10⁹/L with 89% neutrophils and a platelet count of 724 × 10⁹/L, which was consistent with the patient's baseline. The comprehensive metabolic panel showed a creatinine level of 1.4 mg/dL (baseline 0.7 mg/dL), low sodium of 129 mmol/L, elevated potassium of 5.6 mmol/L, low chloride of 92 mmol/L, and a mildly elevated alkaline phosphatase of 133 U/L.

Computed tomography (CT) of the abdomen and pelvis without contrast demonstrated moderate left-sided hydronephrosis and a large heterogeneous structure expanding the left perirenal space, measuring 13.8 x 9.8 x 8.2 cm (Figure 1a,b). This measured intermediate attenuation (30 HU), which could reflect soft tissue, blood products, or other complex fluid. There was an extension of higher-density material into the left paracolic gutter and inferiorly into the left pelvis. Magnetic resonance imaging (MRI) of the abdomen and pelvis was subsequently obtained for further characterization.

**Figure 1:**
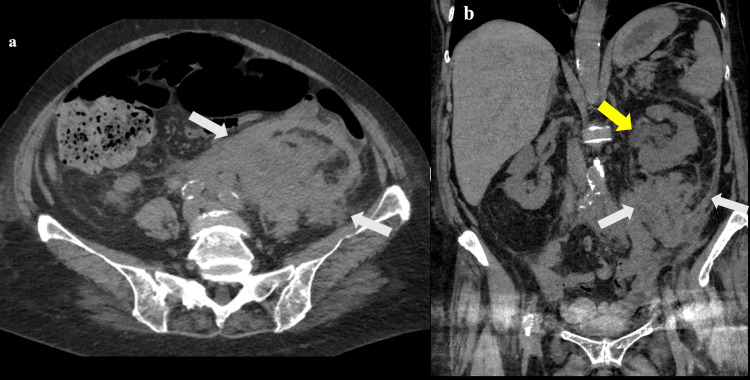
CT axial (a) and coronal (b) images demonstrating a large heterogeneous collection/mass expanding the left perirenal space (white arrows), with extension of higher-density material into the left paracolic gutter and left pelvis (b), causing left-sided hydronephrosis (yellow arrow). Mild-to-moderate hepatomegaly (18.8 cm) is present, with no enlarged lymph nodes and a spleen at the upper limits of normal size. CT: computed tomography.

MRI revealed a large infiltrative, enhancing soft-tissue mass within the left retroperitoneum and pelvis, resulting in moderate left hydronephrosis. The mass demonstrates predominantly intermediate T2 signal with scattered areas of T2 hyperintensity (Figure 2a). Additionally, the mass is predominantly intermediate T1 signal with scattered areas of T1 hypointensity (Figure 2b). Post-contrast T1-weighted imaging demonstrated mild enhancement, favoring a solid soft-tissue mass rather than a complex fluid collection (Figure 2c). Differential diagnoses included infiltrative sarcoma, extramedullary plasmacytoma, and lymphoma.

**Figure 2:**
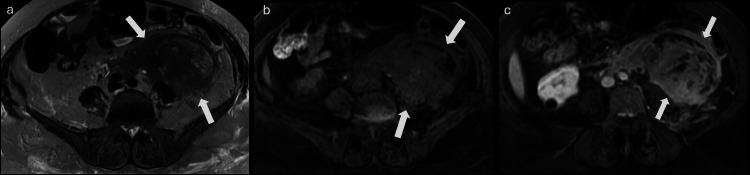
MRI abdomen axial T2 (a), T1(b), and postcontrast T1 (c) images demonstrating a large infiltrative soft tissue mass in the left retroperitoneum and pelvis defined by the white arrows with no enlarged lymph nodes. MRI: magnetic resonance imaging.

To establish the diagnosis, a CT-guided core needle biopsy of the left perirenal lesion was performed, yielding sufficient tissue for histopathologic evaluation and flow cytometric analysis. Histopathologic examination revealed an infiltrate of immature mononuclear cells with ill-defined cytoplasm, prominent nucleoli, and variable cellular clustering. No gland formation, keratinization, spindle cell proliferation, or vascular differentiation was identified. The lesion demonstrated a high proliferative index with Ki-67 of approximately 70-80%, consistent with a highly proliferative malignant neoplasm (Figure 3).

**Figure 3:**
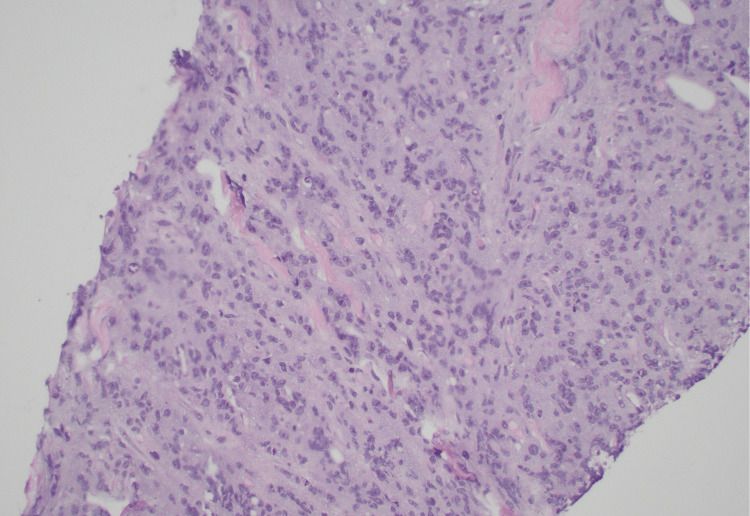
H&E stain at 100× magnification. Mononuclear cells appear immature, with poorly defined cytoplasm, prominent nucleoli, and variable patterns of cellular clustering. There is no evidence of gland formation, keratinization, spindle cell proliferation, or vascular formation.

Immunohistochemical staining demonstrated diffuse positivity for CD43, CD117, CD33, and CD163, supporting myeloid lineage with monocytic differentiation (Figure 4 and Table 1). CD68 positivity further confirmed histiocytic/monocytic differentiation, while scattered CD15 expression was consistent with immature myeloid cells [10]. Myeloperoxidase (MPO) was negative, a finding that can occur in MS with monocytic differentiation as opposed to granulocytic differentiation.

Tumor cells were negative for cytokeratin, lymphoid, melanocytic, and mesenchymal markers, effectively excluding epithelial, lymphoid, melanocytic, and soft tissue sarcomas. Flow cytometry of the mass biopsy was limited by low cellularity and revealed no evidence of a monoclonal B-cell or abnormal T-cell population. In the context of the patient’s known PV, these findings were most consistent with MS with monocytic differentiation.

**Figure 4:**
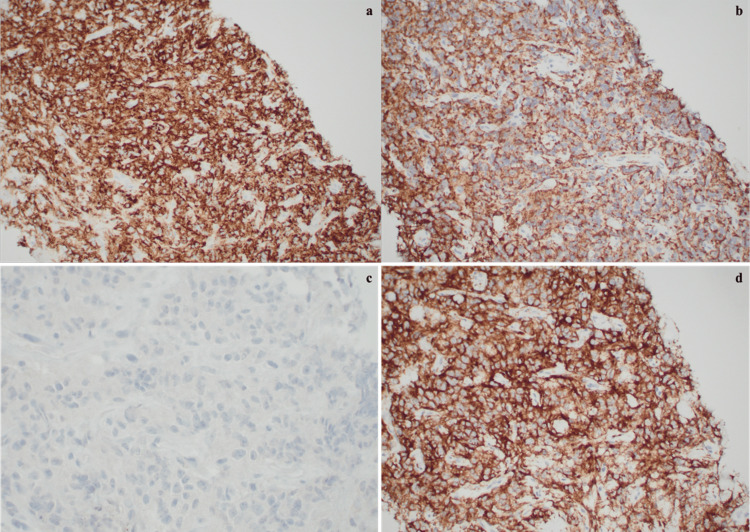
Malignant cells (a) positive for CD43, (b) positive for CD163, (c) negative for Myeloperoxidase (MPO), (d) and positive for CD33.

**Table 1:** Immunohistochemistry panel.

Marker	Result	Interpretation
CD43	Strongly, diffusely positive	Supports hematopoietic/myeloid origin
p16	Strongly, diffusely positive	Non-specific; seen in many malignancies
CD117 (c-KIT)	Positive throughout, moderate intensity	Can be seen in myeloid neoplasms/blasts
CD45 (LCA)	Equivocal	Weak/uncertain leukocyte marker expression
CD68	Positive, variable granular staining	Supports monocytic/histiocytic differentiation
AE1/AE3	Negative	Against carcinoma
CAM5.2	Negative	Against carcinoma
SOX10	Negative	Against melanoma/nerve sheath tumors
CD20	Negative	Against B-cell lymphoma
CD3	Negative	Against T-cell lymphoma
CD34	Negative	No significant blast/stem cell marker expression
CD138	Negative	Against plasma cell neoplasm
CD30	Negative	Against certain lymphomas
S100	Negative	Against melanoma and neural tumors
PAX5	Negative	Against B-cell lineage
SMA	Negative	Against smooth muscle tumors
CD56	Negative	Not supporting NK-cell/neuroendocrine lineage
DOG1	Negative	Against GIST
CD163	Diffusely positive	Strong evidence of monocytic differentiation
CD33	Diffusely positive	Strong myeloid lineage marker
CD15	Scattered positive cells (<10%)	Supports myeloid differentiation
Myeloperoxidase (MPO)	Negative	Commonly negative in monocytic variants

## Discussion

This case highlights the diagnostic challenge of MS and the value of radiology-pathology correlation and multidisciplinary collaboration in achieving an accurate diagnosis. MS is a rare extramedullary manifestation of MPNs, occurring in 2.5-9% of AML patients and less frequently in chronic MPNs such as PV [1,2]. Its variable presentation and frequent absence of bone marrow involvement often delay recognition, particularly in non-leukemic patients. Compared with de novo AML-associated MS, MS arising in the setting of MPN likely reflects a distinct biological process related to clonal evolution and leukemic transformation [8,9]. In MPNs, particularly PV and myelofibrosis, progression to accelerated-phase or blast-phase disease is a significant concern, and extramedullary collections of myeloid blasts may represent one manifestation of this transformation [9]. While de novo AML-associated MS often presents concurrently with or precedes marrow AML, MS linked to MPNs can develop as the disease evolves and should prompt a thorough assessment for blast-phase transformation.

In this case, retroperitoneal and pelvic masses caused non-specific abdominal pain, distension, and hydronephrosis, demonstrating the diverse clinical manifestations of MS. Imaging was essential for localization and assessment of compressive effects, supporting the presence of an ill-defined enhancing soft tissue mass without significant hemorrhagic or necrotic components. Although the size, infiltrative nature, retroperitoneal location, and associated hydronephrosis raise concern for sarcoma, the findings were not specific. Lymphoma, liposarcoma, and extramedullary plasmacytoma can present similarly, particularly as infiltrative retroperitoneal masses causing hydronephrosis [11].

Definitive diagnosis, however, required histopathologic examination and immunophenotyping. Tumor cells expressed CD33, CD117, CD43, CD68, and CD163, consistent with myeloid lineage and monocytic differentiation, while MPO negativity, common in monocytic variants, was observed [11,12]. A high Ki-67 index indicated a highly proliferative tumor, underscoring the need for timely intervention. Assessment of the bone marrow and peripheral blood smear could help further characterize MS and guide management. However, in this case, the patient did not pursue further diagnostic tools and therapeutic management of the disease and elected palliative care and died two weeks after diagnosis.

Management of MS generally involves systemic chemotherapy following AML regimens, as untreated cases frequently progress to AML within 5-12 months [5]. Surgery or radiotherapy is reserved for symptom relief or local control, and stem cell transplantation may be considered in select high-risk patients. Imaging plays an important role in assessing treatment response [5]. Positron emission tomography with 18F-fluorodeoxyglucose (18F-FDG PET) identifies metabolically active tumor regions for radiation planning, while MRI and CT provide complementary anatomic detail for precise tumor delineation and monitoring [13].

The collaboration between hematology-oncology, radiology, and pathology was pivotal, allowing integration of imaging and histologic findings to reach an accurate diagnosis. This case emphasizes several key points, including that clinicians should maintain a high suspicion for atypical masses in patients with MPNs even in the absence of marrow involvement, that early tissue sampling and comprehensive immunohistochemistry are essential, and that multidisciplinary evaluation is crucial to integrate clinical, radiologic, and pathologic data for timely diagnosis.

Limitations

This report is limited by its single-patient design, which affects the broader applicability of the findings. Flow cytometric evaluation of the mass was limited by low cellularity. Bone marrow biopsy and analysis were not conducted because the patient chose to pursue palliative care, leaving the status of marrow involvement uncertain. In addition, cytogenic and molecular studies beyond the known JAK2 mutation were unavailable. Treatment response could not be assessed because the therapeutic approach was not pursued. Furthermore, evaluation of the disease's full extent, clinical progression, and long-term outcomes was limited by the patient's death.

## Conclusions

MS is a rare extramedullary manifestation of myeloid neoplasms that can mimic other malignancies, particularly when arising in deep-seated sites such as the retroperitoneum and pelvis. This case contributes to the limited literature on extramedullary MS in chronic myeloproliferative disorders. Accurate diagnosis requires a combination of imaging, histopathology, and immunophenotypic analysis. Early recognition and prompt systemic therapy are critical to prevent progression to AML and improve patient outcomes. Clinicians should maintain a high index of suspicion for MS in patients with underlying myeloproliferative disorders presenting with atypical masses.
